# Problems, complications, and reinterventions in 4893 onlay humeral lateralized reverse shoulder arthroplasties, a systematic review: part II—problems and reinterventions

**DOI:** 10.1186/s10195-021-00613-8

**Published:** 2021-11-26

**Authors:** Francesco Ascione, Alfredo Schiavone Panni, Adriano Braile, Katia Corona, Giuseppe Toro, Nicola Capuano, Alfonso M. Romano

**Affiliations:** 1 Department of Orthopaedic and Trauma Surgery, Ospedale Buon Consiglio Fatebenefratelli, Via Petrarca 35, 80123 Napoli (NA), Italy; 2 Orthopedics and Sport Medicine Unit, Campolongo Hospital, Salerno, Italy; 3 Dipartimento Multidisciplinare Di Specialità Medico-Chirurgiche Ed Odontoiatriche, Università Degli Studi Della Campania “Luigi Vanvitelli”, Napoli, Italy; 4 Department of Medicine and Health Sciences, Università del Molise, Campobasso, Italy

**Keywords:** Grammont, Results, Revision, Humeral offset, Dislocation, Infection, Radiographic findings

## Abstract

**Background:**

Several modifications to the original Grammont reverse shoulder arthroplasty (RSA) design have been proposed to prevent distinctive issues, such as both glenoid and humeral lateralization. The aim of this systematic review was to determine rates of problems, complications, reoperations, and revisions after onlay lateralized humeral stem RSA, hypothesizing that these are design related.

**Methods:**

This systematic review was performed in accordance with the PRISMA statement guidelines. A literature search was conducted (1 January 2000 to 14 April 2020) using PubMed, Cochrane Reviews, Scopus, and Google Scholar, employing several combinations of keywords: “reverse shoulder arthroplasty,” “reverse shoulder prosthesis,” “inverse shoulder arthroplasty,” “inverse shoulder prosthesis,” “problems,” “complications,” “results,” “outcomes,” “reoperation,” and “revision.”

**Results:**

Thirty-one studies with 4893 RSA met inclusion criteria. The 892 postoperative problems and 296 postoperative complications represented overall problem and complication rates of 22.7% and 7.5%, respectively. Forty-one reoperations and 63 revisions resulted, with overall reoperation and revision rates of 1.7% and 2.6%, respectively.

**Conclusions:**

Problem, complication, and reintervention rates proved acceptable when implanting a high humeral lateralization stem RSA. The most frequent problem was scapular notching (12.6%), and the most common postoperative complication was scapular stress fracture (1.8%). An overall humeral complication rate of 1.9% was identified, whereas no humeral fractures or stem loosening were reported with short stems. Infections (1.3%) were the most common reason for component revision, followed by instability (0.8%).

**Level of evidence:**

Systematic review IV.

## Introduction

Grammont-style reverse shoulder arthroplasty (RSA) provides satisfactory clinical results for several shoulder pathologies [1–4], but this design has been found to have several drawbacks inherent to altered joint biomechanics. Firstly, excessive medialization may lead to a slackening of any intact cuff, which could contribute to undesirable instability, poor restoration, and weakness of internal and external rotation [1, 5]. Secondly, the contour of the shoulder is somewhat altered, and the physiological wrapping angle of the deltoid decreases from 48° to 8°, contributing to instability in association with an insufficient cuff [6–8]. Finally, the 155° neck-shaft angle (NSA) and glenoid medialization leads to peripheral impingement and high rates of scapular notching [1, 4], with the potential for polyethylene wear, glenoid loosening, osteolysis, and tuberosity resorption [8, 9].

Studies have shown that bone lysis, component loosening, and overall complications frequently occur on the humeral side (1.5–10%) [1, 10–13].

Subsequent RSA designs have attempted to address some of these issues by providing more lateralized reconstruction. The following modifications of the stem design have been proposed: (I) a change in the NSA to 145° or 135° to decrease scapular notching, (II) curved and short stems to preserve bone stock and tuberosities, and (III) onlay systems to facilitate conversion from an anatomic arthroplasty. These changes translate into humeral lateralization, which presents several advantages. It restores a more natural anatomical position of the humerus and, therefore, of the lesser and the greater tuberosities, which improves the length/tension of the remaining cuff [1, 14, 15], thus increasing compressive forces on the joint and improving stability [15]. A more lateral position of the greater tuberosity increases the abductor lever arm and the wrapping angle of the deltoid [9], which enhances compressive forces [6, 16, 17].

Medialized implants are now a minority, but the ideal amount of global lateralization and the ideal contribution from the glenoid or the humerus remain unknown. Werthel et al. [18] provided a clear definition of humeral lateralization and values of lateralization in the most commonly used, currently available RSA implants. They concluded that restoring anatomical insertion of remaining cuff, deltoid wrapping angle, and greater tuberosity lateralization corresponds to high humeral offset implants. Lateralization in both the humerus and the glenoid combines the beneficial effects of each lateralization, but the risk is that excessive lateralization may be problematic in smaller patients or in the presence of soft tissue contractures; resultant joint overstuffing may lead to poor motion, polyethylene wear [15, 16], difficulty in joint reduction, nerve stretching, difficulty with the repair of the subscapularis [14, 19], acromial impingement, and/or fractures [20].

The purpose of the present study, therefore, was to perform a systematic review of the published literature to determine the overall rates of problems, complications, reoperations, and revisions after onlay lateralized humeral stem RSA. Definitions of problems/complications and reinterventions (reoperations/revisions) after RSA were based on a previously published review [21] and stated in Table 1.

**Table 1:** Definitions of problems, complications, reoperations, revisions

		Definition	Examples
	Problems	Intraoperative or postoperative event that was not likely to affect the patient’s final outcome	Radiographic scapular notching, hematomas, glenoid or humeral nonprogressive radiolucent lines, heterotopic ossification, scapular spurs, chronic pain and/or stiffness, intraoperative dislocations, intraoperative cement extravasation, or other radiographic findings of the humerus or the eventual glenoid graft
	Complications	Any intraoperative or postoperative event that was likely to have a negative influence on the patient’s final outcome	Fractures, infections, dislocations, nerve palsies, aseptic loosening of humeral or glenoid components, prosthetic components disassociations, or glenoid graft failures
Reinterventions	Reoperations	Intervention requiring any return to the operating room for any reason relating to the shoulder, without replacing humeral/glenoid components	PE insert exchanges, ORIF, debridement, arthroscopy, tendon transfers
	Revisions	Surgeries with total or partial exchange or removal of the components	Stem exchanges, glenoid baseplate/glenosphere exchanges, humeral spacers

*PE* polyethylene insert, *ORIF* open reduction internal fixation

**Table 2:** Demographic data and studies characteristics

First author	Year	Level of evidence	RSA cases	Subgroups	Implanted RSA	Approach	Cemented	Age (years)	Range (± SD)	Follow-up (months)	Range (± SD)	Males	Females
*Franceschetti*	2020	III	59	Not BIO 29; BIO 30	Aequalis Ascend Flex (Tornier—Wright)	DP	N/S	69.7; 70	N/S (± 9.9); N/S (± 7.8)	24.6; 25.2	N/S (± 1.1); N/S (± 1.6)	10; 13	20; 16
*Simovitch*	2019	III	324	Notch 47; not notch 277	Equinoxe (Exactech)	DP	50	72.0	38–89 (± 7)	75.1	60–132 (± 16.9)	N/S	N/S
*Franceschetti*	2019	III	84	SSR 44; not SSR 40	Aequalis Ascend Flex (Tornier—Wright)	DP	N/S	70.18; 69.71	N/S (± 10.63); N/S (± 6.14)	15.9; 16.92	N/S (± 1.29); N/S (± 1.92)	10; 11	34; 29
*Choi*	2019	IV	38		Comprehensive Reverse (Biomet)	DP	0	73	63–83	24	12–53	6	32
*Aibinder*	2019	IV	65		Comprehensive Micro Stem (Zimmer-Biomet)	DP	0	N/S	N/S	45.6	N/S	N/S	N/S
*Raiss*	2019	IV	77		Aequalis Ascend Flex (Tornier—Wright)	DP	N/S	72	50–91	28	24–48	N/S	N/S
*Matsuki*	2018	III	552	Small 130; Average 384; Tall 38	Equinoxe (Exactech)	N/S	N/S	74; 72; 72	50–85; 52–93; 59–84	41; 37; 37	24–94; 24–97; 24 -98	4; 178; 37	126; 206; 1
*Merolla*	2018	III	38		Aequalis Ascend Flex (Tornier—Wright)	DP	3	74.7	55–91	29.1	24–31	13	25
*Ascione*	2018	IV	485	Sc Fr 21; not Sc Fr 84	Aequalis Ascend Flex (Tornier—Wright)	DP	N/S	72.6; 72.3	N/S (± 7.1); N/S (± 7.6)	16.3; 16.5	6.5; 9.1	6; 24	15; 60
*Alentorn-Geli*	2018	III	16	All B2 glenoids	Comprehensive Reverse (Zimmer-Biomet)	DP	N/S	72.5	N/S (± 5.4)	35.1	N/S (± 14.2)	N/S	N/S
*Werner BC*	2018	III	109	SSR 71; not SSR 38	Aequalis Ascend Flex (Tornier—Wright)	N/S	N/S	71.1; 70.7	N/S (± 10.7); N/S (± 8.6)	24; 25.2	N/S (± 18); N/S (± 18)	28; 15	43; 23
*Zilber*	2018	IV	35		Aramis (3S Ortho)	DP	0	73	45–86	24	N/S	7	28
*Werner BS*	2017	IV	56		Aequalis Ascend Flex (Tornier—Wright)	DP	9	74.6	56–91	30.1	24–44	15	41
*Mollon*	2017	III	476		Equinoxe (Exactech)	N/S	N/S	72.5	53–90	38	22–93	164	312
*Romano*	2017	IV	112		Equinoxe (Exactech)	DP	0	72.2	60–87	29.2	12–36	29	83
*Kennon*	2017	III	318	Bp sup screw 206; Bp inf screw 112	Equinoxe (Exactech)	DP	N/S	65.3; 65	N/S (± 11); N/S (± 10.8)	N/S	N/S	85; 44	121; 68
*Ascione*	2017	IV	100		Aequalis Ascend Flex (Tornier—Wright)	DP	20	73.4	55–91	32.6	24–44	28	72
*Lädermann*	2017	II	35		Aequalis Ascend Flex (Tornier—Wright)	DP 18, SSCS 17	N/S	78	N/S (± 7)	18	12–46 (± 11)	8	27
*Schnetzke*	2017	IV	25		Aequalis Ascend Flex (Tornier—Wright)	DP	0	N/S	N/S	25	20–35	5	20
*Vourazeris*	2017	III	202	SSR 86; not SSR 116	Equinoxe (Exactech)	DP	N/S	71.6; 71.1	N/S	39.6; 37.2	N/S	N/S	N/S
*Grubhofer*	2017	IV	44		Reverse Anatomical Shoulder System (Zimmer)	N/S	31	68	30–86	46	24–108	32	12
*Hurwit*	2017	III	40		Comprehensive Reverse (Biomet)	N/S	N/S	68.6	N/S (± 7.6)	32.2	N/S (± 14.9)	16	24
*Friedman*	2017	III	591	SSR 340; not SSR 251	Equinoxe (Exactech)	N/S	N/S	72.2; 72.9	N/S	37.3; 35.7	N/S	119; 105	221; 146
*Mollon*	2016	III	297		Equinoxe (Exactech)	N/S	N/S	72	50–88	39	24–91	99	191
*Jones*	2016	IV	44	All glenoid autograft; allograft	Equinoxe (Exactech)	DP	N/S	69.1	N/S (± 7.4)	40.6	16	20	24
*Dezfuli*	2016	III	36	FrS 24; HA Rev 12	Equinoxe (Exactech)	DP	36	73; 66; 66	N/S	36; 34; 24	N/S	4; 2; 2	9; 10;9
*Katz*	2016	IV	140		Arrow (FH Orthopedics)	25 DP, 115 SL	34	72	52–90 (± 6.91)	45	24–120	34	100
*King*	2015	III	83		Equinoxe (Exactech)	DP	32	72	55–93	42	N/S	32	51
*Gilot*	2015	III	292	Cem 177; 115 UnC	Equinoxe (Exactech)	DP	177	N/S	N/S	39.76	N/S	N/S	N/S
*Giuseffi*	2014	IV	44		Comprehensive Reverse (Biomet)	DP	0	76	59–92	27	24–40	15	29
*Valenti*	2011	IV	76		Arrow (FH Orthopedics)	SL	6	73	52–90	44	24–60	18	58
*TOTAL*	…	…	4893	SSR 541/986	Equation 68%, AFl 19.6%, Com 6.4%, Arr 4.4%, Inv 0.9%, Ara 0.7%	2593 DP + 191 SL/2784	398/1508	4511	…	4195	…	1238	2286
*Mean/%*				SSR 54.9%		DP: 93.1% SL: 6.9%	26.4%	71.5	…	38.6	…	35.1%	64.9%

*RSA* reverse shoulder arthroplasty, *SD* standard deviation, *Notch* scapular notching, *BIO* bony increased-offset reverse shoulder arthroplasty, *SSR* subscapularis tendon repaired, *Sc Fr* scapular fractures, *Bp sup screw* metaglene baseplate superior screw used, *Bp inf screw* metaglene baseplate inferior screw used, *FrS* fractures sequelae, *HA Rev* hemiarthroplasty revisions, *Cem* cemented stem, *UnC* uncemented stem, *DP* deltopectoral, *SL* superolateral, *SSCS* subscapularis and deltoid sparing, *N/S* not stated, *Equ* Equinoxe (Exactech), *AFl* Aequalis Ascend Flex (Tornier—Wright), *Com* Comprehensive Reverse (Biomet), *Arr* Arrow (FH Orthopedics), *Inv* Reverse Anatomical Shoulder System (Zimmer), *Ara* Aramis (3S Ortho)

**Table 3:** Etiologies

First author	Year	CTA	OA	FrS	MRCT	Rev	IA	AvN	AcF	InstA
Franceschetti	2020	38								
Simovitch	2019	–	–				–			
Franceschetti	2019	–	–							
Choi	2019	30	3		5					
Aibinder	2019	33	25	1			4	2		
Raiss	2019	–	–			–				
Matsuki	2018	258	207		61		11	9		
Merolla	2018	59								
Werner BC	2018	–	–							
Zilber	2018	19	10	3				1	2	
Werner BS	2017	44		5	5		2			
Mollon	2017	–	–	–	–					
Romano	2017	–	–	–	–					
Kennon	2017	–		–	–	–				
Ascione	2017	46	28	8	14		2			2
Lädermann	2017	10		8	17					
Schnetzke	2017	17	2	5						
Vourazeris	2017	–	–		–		–	–		
Grubhofer	2017			44						
Hurwit	2017	–	–			–			–	
Friedman	2017	–	–		–		–			
Mollon	2016	–	–	–	–		–	–		
Jones	2016		–			–				
Dezfuli	2016			24		12				
Katz	2016	–			–					
King	2015	–	–		–					
Giuseffi	2014	33		2			3	6		
Valenti	2011	–	–	15	–	25	2		6	2
TOTAL		587	275	115	102	37	24	18	8	4
%		50.2%	23.5%	9.8%	8.7%	3.2%	2.1%	1.5%	0.7%	0.3%

*CTA* cuff tear arthropathy, *OA* glenohumeral osteoarthritis, *FrS* fractures sequelae, *MRCT* massive rotator irreparable cuff tears, *Rev* revisions, *IA* inflammatory arthritis, *AvN* avascular necrosis of the humeral head, *AcF* acute fractures, *InstA* instability arthropathy

Studies that did not declare number of cases for each etiology

Alentorn-Geli^2^, Ascione ^6^, and Gilot ^22^ did not declare etiologies

**Table 4:** Problems and radiographic findings

Postoperative Problems	Cases (No.)	%
Scapular notching Stage 1 Stage 2 Stage 3 Stage 4	411 251 82 36 4	12.6 67.3 22 1.2 0.1
Humeral radiolucent lines	151	5.8
Stress shielding Proximal humeral remodeling Tuberosities resorption Calcar osteopenia Lateral cortical osteopenia Spot welds Condensation lines	106 31 4 24 75 24 21	29.4
Glenoid grafts Radiolucent lines Not fully incorporated Failure	63 8 2	42.9 5.4 1.4
Heterotopic ossification	49	
Scapular spur	41	
Chronic pain	26	
Shoulder stiffness	20	
Glenoid radiolucent lines	13	0.8
Deltoid strain	1	
Total	892	22.7

**Table 5:** Postoperative complications, reoperations, and revisions

Postoperative complications	Cases (No.)	%	Reoperations	Revisions
Instability	29	0.8	7 thicker PE replacement	3
Acromion and scapular spine fractures Type I Type II–III	77 14 41	1.8	10 ORIF 1 arthroscopic acromioplasty	1 (+ ORIF)
Humeral fractures	50	1.4	8 ORIF 1 acromioplasty	5 (+ ORIF)
Infections	47	1.3	3 PE change + debridement 2 debridements	18
Aseptic glenoid loosening	39	1.1		7
Humeral loosening	19	0.5		6
Glenoid/humeral disassembly	16		5 PE change	16
Neurologic complications	12	0.4		1
PE wear	2		2 PE change	
Unspecified implant failures	2			2
Pulmonary embolism	2	0.05		
Glenoid graft failure	2			2
Hematoma	1			1
Draining axilla folliculitis	1		1 debridement	
Shoulder pseudoparalysis	1		1 latissimus dorsi tendon transfer	
Total	296	7.5	41 (1.7%)	63 (2.6%)

*PE* polyethylene insert, *ORIF* open reduction internal fixation

It was hypothesized that emerging reinterventions, problems, and complications are peculiar to new design prostheses and their significance differs from that of Grammont-style RSA. In this part, a systematic review of problems and reinterventions was performed [22].

## Materials and methods

This systematic review was conducted according to the guidelines of the preferred reporting items for systematic review and meta-analysis (PRISMA) statement (http://prisma-statement.org).

### Search strategy

A systematic review of the available literature was performed using synonymous or related expressions for the terms “reverse shoulder arthroplasty,” “reverse shoulder prosthesis,” “inverse shoulder arthroplasty,” “inverse shoulder prosthesis,” “problems,” “complications,” “results,” “outcomes,” “reoperation,” and “revision,” in several combinations. The following databases were assessed: PubMed, Cochrane Reviews, Scopus, and Google Scholar. The search was performed from 1 January 2000 to 14 April 2020. All peer reviewed journals were considered, and randomized controlled trials (RCTs), prospective trials (PRO), and retrospective studies (RE) were included. The search was limited to papers in the English language. Two authors (A.B. and G.T.) independently screened the titles and abstracts, and subsequently performed a full-text selection of the articles resulting from the search. All references of the included studies were subsequently searched manually to identify any additional articles that may not have been captured in the initial search. In the event of disagreement, a consensus was reached by discussion, with the intervention of the senior author (F.A.) when necessary.

### Study selection

For the aforementioned aim, the implants included were derived from the study of Werthel et al. [18], with prostheses of minimum 10 mm humeral lateralization compared with Grammont-style RSA, resulting in a 10–14.7 mm lateral offset range, 135–145° NSA, and all onlay designs.

To be considered eligible for inclusion, studies needed to: (1) include patients who had undergone an onlay humeral lateralized RSA; (2) report data on problems, complications, reinterventions, and revisions with declared implants; (3) be a published RCT, RE study, or PRO trial.

Studies were excluded if: (1) the articles were not in English; (2) it was impossible to extrapolate or calculate the necessary data from the published results; (3) it was a review article or technical note; (4) they involved animal experiments or in vitro trials; (5) they focused exclusively on acute fractures, revisions, or tumor surgery series; (6) they presented a heterogeneous use of Grammont and humeral lateralized arthroplasties in a single cohort.

### Level of evidence

The Oxford Levels of Evidence, as produced by the Oxford Centre for Evidence-Based Medicine, was used to categorize methodological quality (http://www.cebm.net/ocebm-level-of-evidence/). This tool classifies systematic randomized clinical trials and inception cohort studies as level II evidence, cohort studies, or control arm of randomized trials as level III evidence, and case series or case–control studies or poor-quality prognostic cohort studies as level IV evidence.

### Methodological quality assessment

Methodological evaluation was performed according to the MINORS evaluation [23] which was specifically created to evaluate the quality of nonrandomized surgical studies. The checklist includes 12 items, with the last 4 specific to comparative studies. Scoring was as follows: 0, not reported; 1, reported but poorly done and/or inadequate; and 2, reported, well done, and adequate. The highest overall score was 16 for noncomparative studies and 24 for comparative studies.

### Data extraction

Two authors (A.B. and G.T.) extracted data from all selected original articles; this procedure was repeated by another author (K.C.). If no agreement could be reached, the senior author (F.A.) was consulted. Data were extracted from each included article and entered into a spreadsheet for analysis. The following information was extracted from all studies systematically using a table template: author, date and journal of publication, study design and level of evidence, patient demographics (number of shoulders enrolled, gender, age, and follow-up), prosthetic implant used, surgical approach, diagnosis leading to RSA, intraoperative complications, postoperative problems/complications, reoperations, and revisions. Definitions of problems/complications and reoperations/revisions were based on a previously published review [21], with certain modifications (Table 1). A 0% rate of problem or reintervention was reported whenever the authors stated that none of their patients had that problem or complication, whereas the value was left as unreported when authors did not mention the problem or complication.

## Results

### Literature search

**Fig. 1:**
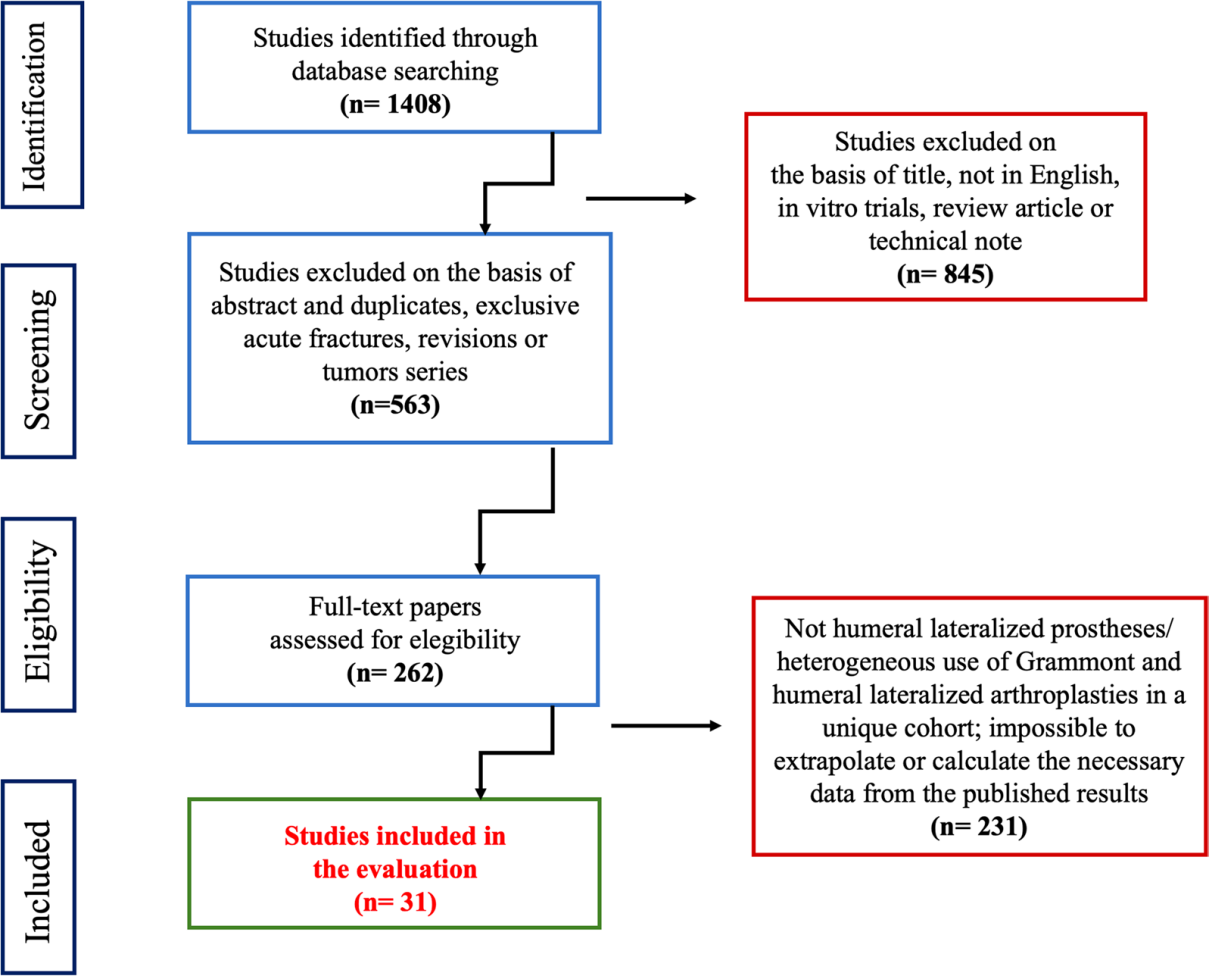
Articles selection process of the review

The initial search resulted in 1408 articles. The abstracts of these studies were reviewed to determine the applicability to the present study as determined by the inclusion and exclusion criteria, including a worksheet adapted from evidence-based guides (Fig. 1).

A total of 4893 RSA were included from 31 studies [14, 19, 20, 24–51].

Average MINORS scores were 13.7/16 for noncomparative studies and 19.4/24 for comparative studies, thus demonstrating an acceptable study quality level.

### Demographic data, surgical technique, and etiology

Demographics of the reviewed cohort and follow-up, including study design, level of evidence, surgical information, and etiology are summarized in Table 2. Twenty-five studies declared the gender of patients, and mean postoperative follow-up was 38.6 months.

The indication for implanting an RSA was stated in 28 studies (4100 cases), but 12 studies did not state the number of cases for each etiology and, in one, some cases were unstated [48], leaving a total of 1170 arthroplasties with definite pathologies.

The most frequent surgical indications were cuff tear arthropathy (CTA) in 587 shoulders (50.2%). Details of the analyzed study etiologies are reported in Table 3.

## Results

Of the 3926 cases, the 892 postoperative problems represented an overall rate of 22.7%. One hundred and four reinterventions resulted in 2435 prostheses, 41 reoperations, and 63 revisions, with rates of 1.7% and 2.6%, respectively.

Problems/radiographic findings and reoperations/revisions are summarized in Tables 4 and 5, respectively.

### Postoperative problems and radiographic findings

The most common postoperative problem was radiographic scapular notching, which was present in 411 of 3268 cases (12.6%). The Sirveaux classification [2] was available for 2883 cases (88.2% of reported scapular notching), of which 251 were stage 1 (67.3%), 82 stage 2 (22%), 36 stage 3 (1.2%), and 4 stage 4 (0.1%).

Grubhofer et al. [33] recorded a high rate (93%) of scapular notching, with 41 out of 44 shoulders operated after fracture sequelae of failed osteosynthesis intervention. This was the only study that reported stage 4 notching (four cases). On exclusion of the aforementioned article, the mean incidence of scapular notching falls to 11.5% (370 cases of 3224 RSA).

The next most frequent postoperative problem was radiolucent lines around the humeral component, with no clinical effect (151 cases of 2618 RSA) and a mean incidence of 5.8%.

Most cases (143 of 2068 RSA; 6.9%) were in Equinoxe Reverse prosthesis, whereas no cases of radiolucent lines were reported in implanted Comprehensive Reverse or Aramis Reverse prostheses.

Lucent lines around the glenoid were rare (0.8%), mentioned in 13 of 1683 cases, and not requiring treatment.

Six studies [24, 32, 41, 44–46] investigated radiological changes around the lateralized stems, totaling 361 cases: some manifestation of humeral stress shielding (such as proximal humeral remodeling/tuberosities resorption, cortical osteopenia/calcar resorption, spot welds, or condensation lines) was found in 106 shoulders (29.4%); more specifically, 31 postoperative cases of proximal humeral remodeling/4 tuberosities resorptions, 24 shoulders with calcar osteopenia, 75 cases of lateral cortical osteopenia, 24 spot welds, and 21 condensation lines.

A scapular spur was found in 41 cases and a glenohumeral heterotopic ossification in 49.

However, since few studies reported the aforementioned problems, they may be underestimated. In the studies dealing with glenoid grafts (147 cases) [26, 29, 35, 41], radiographic lucent lines were found around 63 glenoid grafts, suggesting a partial incorporation; in 8 cases the graft was not incorporated and in 2 they had failed, producing loose baseplates requiring revision surgery.

Overall, 50 other problems (2%) were indicated in 8 studies, in a total of 2464 prostheses (Table 4): 26 patients with chronic pain, 20 cases of shoulder stiffness, 1 case of deltoid muscle strain, 1 RSA with a hematoma, 1 case of shoulder pseudoparalysis, and 1 case of draining axilla folliculitis. The three last cases mentioned required reintervention.

### Reoperations and revisions

The most common reoperation was open reduction and internal fixation after humeral or scapular fracture, 18 cases (0.7%; 10 for scapular fracture, 8 for humeral fractures).

The PE liner was changed in 17 cases: 6 due to PE dissociation, 1 due to PE wear, and 7 thicker PE changes were performed after RSA dislocation. Infections that did not require revision of components were treated through 3 PE insert changes and debridements, 1 arthroscopic debridement, and 1 open debridement. Other reoperations comprised 1 case of debridement for draining axilla folliculitis, 1 reattachment of the greater tuberosity combined with a latissimus dorsi tendon transfer for a shoulder pseudoparalysis, 1 arthroscopic acromioplasty after scapular fracture and malunion with secondary impingement, and 1 open acromioplasty after greater tuberosity impingement in a humeral fracture.

Revision surgeries were performed most commonly for infection, in 18 cases (0.7%): 3 unspecified revisions for infection, 8 one-stage procedures, and 7 two-stage revision surgeries (Table 5).

An aseptic component loosening requiring revision, both glenoid and humeral, was reported in 13 cases (6 humeral loosening, 7 glenoid loosening) and 5 revisions as a consequence of humeral periprosthetic fractures. The glenospheres or the PE separated from either the metal baseplate or the humeral stem, respectively, in 10 cases of a particular first-generation prosthetic design. These were all revised[36, 48].

The instability complicated prostheses necessitated revision in three cases (one unspecified procedure, one humeral and PE change, and one change to thicker PE insert, humeral tray, and larger glenosphere).

Other complications requiring revision included one hematoma, one type III spine fracture (revision and simultaneous ORIF), and one postoperative brachial palsy, neurolysis, and revision with a shorter stem replacement.

## Discussion

The present review showed fewer problems and less reintervention rates when implanting a high humeral lateral offset (range 10–14.7 mm), 135°–145° NSA, and onlay system RSA, compared with Grammont-style designs [18].

The global rates for problems, reoperations, and revisions after onlay humeral lateralized RSA were 22.7%, 1.7%, and 2.6%, respectively, at 3 years mean follow-up. To our knowledge, no studies in the literature have thoroughly investigated the topic of this systematic review.

The principal finding was that prostheses with a lateralized humeral stem resulted in lower rates in the distinctive problems of Grammont-style RSA, which were reported as much higher in previous literature [1–4, 10–13, 21, 52–56].

Scapular notching was the most frequently reported problem after the analyzed RSA, even though it did not achieve the 35–96% rate that had previously been reported for RSA with a medialized center of rotation[1–4, 10–13, 21, 52–55]. Notching emerged with a 12.6% incidence and almost 90% of cases pertained to stage 1 or 2 scapular notching.

Nevertheless, scapular notching remains a concern of relevance in certain etiologies, such as fracture sequelae [57]. Grubhofer et al. [33] studied 44 shoulders operated on after failed osteosynthesis of proximal humeral fractures; 41 out of 44 cases (93%) were affected by scapular notching, and it was the only study that reported a stage 4 notching (4 cases). Indeed, the mean incidence of scapular notching decreased to 11.5% on exclusion of this article.

Although no studies have actually proven scapular notching as a cause of glenoid loosening, it has an implicit negative impact on clinical outcome scores, complications, revisions, and humeral radiolucent lines [47, 52].

In the past, the safest methods of preventing notching were considered to be inferior positioning/tilting of the glenoid baseplate, larger size glenospheres, and the use of bone or metal increased offset glenoids [54, 55, 58].

With decreasing scapular notching and increasing impingement-free motion in glenoid lateralized RSA, the force required for the deltoid to perform abduction and acromial stress increases [16]. Therefore, the baseplate is subjected to substantial shear forces, and a significantly higher risk of glenoid radiolucency lines and aseptic glenoid loosening subsequently needing revision has been reported [13, 21, 54]. In the present review, lucent lines around the glenoid were an uncommon problem (0.8%), without any clinical effect. Out of 13 shoulders, 6 cases of baseplate with radiolucent lines resulted from a study that included only problematic preoperative glenoids with severe bone loss, and consequently, 44 bone autografts/allografts [35], suggesting that glenoid lateralization is limited by bone erosion, inclination, or retroversion. In contrast, another article studied a B2 glenoids cohort with RSA and, at a 3-year mean follow-up, no cases of glenoid lucent lines or loosening were found.

More recently, attention has switched to lateralizing the humeral side, achieved by various means with several aforementioned advantages. Firstly, the stem may be modified from straight to curved [26]. Secondly, the humeral bearing may rest on the humeral osteotomy in the onlay system, lateralizing the humerus by displacing the stem away from the glenosphere. All implants evaluated in this study had an onlay design, thus preserving metaphyseal bone, ensuring ease of conversion, and providing additional modularity of the insert for NSA [46]. Modification of the NSA from 155° to 145° or 135° has been described as a cause and/or a means of humeral lateralization, with no increase in instability [59].

The effects of humeral lateralization may be observed in radiological changes around the stem, from manifestations of stress shielding, bone remodeling, and resorption to humeral radiolucent lines. The latter proved to be the second most frequent problem, reported with a prevalence of 5.8% in 151 shoulders, with at least one lucent line in one stem zone, particular to some designs (Equinoxe, about 7%) and totally absent in others (Comprehensive and Aramis). No clinical effects were demonstrated following these radiological findings, nor in the six studies that investigated proximal humeral remodeling, cortical, calcar or tuberosities osteolysis/osteopenia, condensation lines and spot welds: about 30% of shoulders were found to have at least one of the cited detections, and particular studies were conducted into short stems [14, 20, 24, 26, 29, 39, 41, 44, 46, 50]. Concerns exist that the malalignment of the component (related to stem recent design and consequently to the surgeons’ experience) could potentially produce bone adaption phenomena or suboptimal bone ingrowth [24, 44, 46, 60].

In contrast, postoperative humeral fractures were generally treated conservatively (in 74% of cases) and in transverse or spiral fractures with minimal displacement, splint immobilization can ensure consolidation in 3–6 months. The available literature suggests treating displaced transverse fractures through plate osteosynthesis, with or without autologous bone graft [10] and, in the case of associated loosening and/or instability, long stem implant revision is required [61]: 16% and 10% of fractures in this study were treated through osteosynthesis and stem revision, respectively. However, in shoulders with thin cortical bone in the proximal humerus and tuberosities, extensive cementing around the prosthesis in the area of the tuberosities to prevent component rotation may be preferable to revision [22, 59, 62].

The most common reoperation was open reduction and internal fixation after periprosthetic fracture (0.7%), with 10 reinterventions for a scapular fracture and 8 for a humeral fracture.

Fixation of scapular fractures is challenging because of mechanical conditions brought about by the combination of deltoid muscle distraction forces and osteoporotic bone. In addition, fracture management guidelines are not clearly identified [20, 37, 41]. The included studies stated reintervention for scapular fracture in 14% of cases (10 ORIF and 1 revision and simultaneous ORIF); the remaining cases were managed through conservative treatment.

Infections remained the most common reason for component revision, and the incidence of infection may increase at longer follow-up periods. Treatment of acute infection using antibiotics and debridement with retention, irrigation, and suction, complemented by intravenous antibiotics, is a choice for infections with symptoms at less than 3 weeks in a stable prosthesis and no growth on preoperative cultures [61, 63]. This regimen is ineffective in chronic or late infections requiring revision, with no clear tendency to use a one-stage or two-stage procedure.

Postoperative dislocations subsequent to RSA had a prevalence of 0.8%, suggesting that one of the main purposes of humeral lateralized design, the increase of prosthetic construction stability, had been obtained. Grammont-style RSA large series and reviews showed a higher instability incidence, ranging from 3% to 14% [2–4, 11, 13, 21, 53, 64], although studies included only glenoid lateralized designs [55].

Although the treatment of prosthetic instability can be conservative, revision surgery may be required in recurrent dislocations and those occurring in the first few months. Shoulder stability can be obtained through prosthetic revision but renders lower functional results [64]. In the present study, the second recurring reintervention was PE liner change, both after dislocation and infection, in association with open debridement.

Fortunately, neurologic injuries were very rare (0.4%) and had an effect only in cases of incomplete recovery: only one case of the 3114 RSA included led to humeral component revision to resolve a brachial plexus palsy. Other brachial plexus/axillary nerve palsies recovered spontaneously, and no reports of radial or ulnar nerve problems were found.

Postoperative glenoid or humeral disassembly and polyethylene disassociations were infrequent and only mentioned as a problem related to the design of the Arrow prosthesis used before 2005, which was resolved after a new implant design [36, 48].

### Limitations and strengths

Our investigation is an up-to-date systematic review of the literature, which considers implants categorized as humeral onlay lateralized design as compared with original Grammont RSA [18]. To date, no studies have thoroughly investigated this particular design in a systematic review.

However, we have identified some study limitations. Firstly, given that almost all the studies included were therapeutic case series, this study corresponds to an indirect level III–IV of evidence, and further research is needed to investigate at level I or II comparative studies.

Second, the definition of problem or complication differs significantly among the studies. Similarly, some authors used the terms “reoperation” and “revision” interchangeably. This may decrease the accuracy of the comparison between the results of this study and those in existing literature. This issue does not affect the accuracy of the analysis in this study, as special attention was paid when collecting data from all the included studies to adequately classify reoperations, revisions, problems, and complications according to the aforementioned definitions, and provide adequate homogenization.

Thirdly, we intentionally excluded studies regarding revision cases only or only proximal humerus acute fractures: this may result in underestimated rates of problems/complications and reinterventions, but this decision [3, 11, 13, 21] was made with the purpose of analyzing this particular design in the most common indications for RSA and high rates of complications/revisions mainly related to revision/fracture surgery, and not the RSA itself. Most of the included cohorts report heterogeneous etiologies.

Finally, there are a huge number of factors that can influence the rates considered, and these are not well controlled for in the existing evidence, these factors include the following: the length of follow-up, the surgeon’s experience, different rehabilitation protocols, the type of glenosphere (eccentric or concentric, medialized or lateralized), humeral version, degree of bone stock and glenoid erosion, the use of cement, or previous surgeries.

## Conclusions

Rates of problems, reoperations, and revisions may be regarded as acceptable (22.7%, 1.7%, and 2.6%, respectively) when implanting a humeral lateralized stem, 135–145° NSA, and onlay RSA. Low overall rates of scapular notching (12.6%), which remains the most frequent problem, and instability (0.8%) were reported. Infections (1.3%) were the most common reason for component revision, and this rate may well increase with a longer follow-up period.

## Data Availability

Not applicable.

## References

[1] Boileau P, Watkinson DJ, Hatzidakis AM, Balg F (2005). Grammont reverse prosthesis: design, rationale, and biomechanics. J Shoulder Elbow Surg.

[2] Sirveaux F, Favard L, Oudet D (2004). Grammont inverted total shoulder arthroplasty in the treatment of glenohumeral osteoarthritis with massive rupture of the cuff. Results of a multicentre study of 80 shoulders. J Bone Joint Surg Br.

[3] Wall B, Nové-Josserand L, O’Connor DP (2007). Reverse total shoulder arthroplasty: a review of results according to etiology. J Bone Jt Surg.

[4] Werner CML (2005). Treatment of painful pseudoparesis due to irreparable rotator cuff dysfunction with the delta III reverse-ball-and-socket total shoulder prosthesis. J Bone Jt Surg Am.

[5] Rojas J, Joseph J, Liu B (2018). Can patients manage toileting after reverse total shoulder arthroplasty? A systematic review. Int Orthop.

[6] Henninger HB, Barg A, Anderson AE (2012). Effect of lateral offset center of rotation in reverse total shoulder arthroplasty: a biomechanical study. J Shoulder Elbow Surg.

[7] Gutierrez S, Levy JC, Lee WE (2007). Center of Rotation affects abduction range of motion of reverse shoulder arthroplasty.. Clin Orthop PAP.

[8] Roche CP, Stroud NJ, Martin BL (2013). The impact of scapular notching on reverse shoulder glenoid fixation. J Shoulder Elbow Surg.

[9] Routman HD, Flurin P-H, Wright TW (2015). Reverse Shoulder arthroplasty prosthesis design classification system. Bull Hosp Jt Dis.

[10] Boileau P (2016). Complications and revision of reverse total shoulder arthroplasty. Orthop Traumatol Surg Res.

[11] Farshad M, Gerber C (2010). Reverse total shoulder arthroplasty—from the most to the least common complication. Int Orthop.

[12] Melis B, DeFranco M, Lädermann A (2011). An evaluation of the radiological changes around the Grammont reverse geometry shoulder arthroplasty after eight to 12 years. J Bone Joint Surg Br.

[13] Wright T, Alentorn-Geli E, Samitier G, Torrens C (2015). Reverse shoulder arthroplasty. Part 2: systematic review of reoperations, revisions, problems, and complications. Int J Shoulder Surg.

[14] Franceschetti E, de Sanctis EG, Ranieri R (2019). The role of the subscapularis tendon in a lateralized reverse total shoulder arthroplasty: repair versus nonrepair. Int Orthop.

[15] Langohr GDG, Giles JW, Athwal GS, Johnson JA (2015). The effect of glenosphere diameter in reverse shoulder arthroplasty on muscle force, joint load, and range of motion. J Shoulder Elbow Surg.

[16] Giles JW, Langohr GDG, Johnson JA, Athwal GS (2015). Implant design variations in reverse total shoulder arthroplasty influence the required deltoid force and resultant joint load. Clin Orthop Relat Res.

[17] Costantini O, Choi DS, Kontaxis A, Gulotta LV (2015). The effects of progressive lateralization of the joint center of rotation of reverse total shoulder implants. J Shoulder Elbow Surg.

[18] Werthel J-D, Walch G, Vegehan E (2019). Lateralization in reverse shoulder arthroplasty: a descriptive analysis of different implants in current practice. Int Orthop.

[19] Vourazeris JD, Wright TW, Struk AM (2017). Primary reverse total shoulder arthroplasty outcomes in patients with subscapularis repair versus tenotomy. J Shoulder Elbow Surg.

[20] Ascione F, Kilian CM, Laughlin MS (2018). Increased scapular spine fractures after reverse shoulder arthroplasty with a humeral onlay short stem: an analysis of 485 consecutive cases. J Shoulder Elbow Surg.

[21] Zumstein MA, Pinedo M, Old J, Boileau P (2011). Problems, complications, reoperations, and revisions in reverse total shoulder arthroplasty: a systematic review. J Shoulder Elbow Surg.

[22] Ascione F, Schiavone Panni A, Braile A (2021). Problems, complications, and reinterventions in 4893 onlay humeral lateralized reverse shoulder arthroplasties: a systematic review (part I—complications). J Orthop Traumatol.

[23] Slim K, Nini E, Forestier D (2003). Methodological index for non-randomized studies (minors): development and validation of a new instrument. ANZ J Surg.

[24] Aibinder WR, Bartels DW, Sperling JW, Sanchez-Sotelo J (2019). Mid-term radiological results of a cementless short humeral component in anatomical and reverse shoulder arthroplasty. Bone Jt J.

[25] Alentorn-Geli E, Wanderman NR, Assenmacher AT (2018). Anatomic total shoulder arthroplasty with posterior capsular plication versus reverse shoulder arthroplasty in patients with biconcave glenoids: A matched cohort study. J Orthop Surg.

[26] Ascione F, Bugelli G, Domos P (2017). Reverse shoulder arthroplasty with a new convertible short stem: preliminary 2- to 4-year follow-up results. J Shoulder Elb Arthroplasty.

[27] Choi S, Bae J-H, Kwon YS, Kang H (2019). Clinical outcomes and complications of cementless reverse total shoulder arthroplasty during the early learning curve period. J Orthop Surg.

[28] Dezfuli B, King JJ, Farmer KW (2016). Outcomes of reverse total shoulder arthroplasty as primary versus revision procedure for proximal humerus fractures. J Shoulder Elbow Surg.

[29] Franceschetti E, Ranieri R, Giovanetti de Sanctis E (2020). Clinical results of bony increased-offset reverse shoulder arthroplasty (BIO-RSA) associated with an onlay 145° curved stem in patients with cuff tear arthropathy: a comparative study. J Shoulder Elbow Surg.

[30] Friedman RJ, Flurin P-H, Wright TW (2017). Comparison of reverse total shoulder arthroplasty outcomes with and without subscapularis repair. J Shoulder Elbow Surg.

[31] Gilot G, Alvarez-Pinzon AM, Wright TW (2015). The incidence of radiographic aseptic loosening of the humeral component in reverse total shoulder arthroplasty. J Shoulder Elbow Surg.

[32] Giuseffi SA, Streubel P, Sperling J, Sanchez-Sotelo J (2014). Short-stem uncemented primary reverse shoulder arthroplasty: clinical and radiological outcomes. Bone Jt J.

[33] Grubhofer F, Wieser K, Meyer DC (2017). Reverse total shoulder arthroplasty for failed open reduction and internal fixation of fractures of the proximal humerus. J Shoulder Elbow Surg.

[34] Hurwit DJ, Liu JN, Garcia GH (2017). A comparative analysis of work-related outcomes after humeral hemiarthroplasty and reverse total shoulder arthroplasty. J Shoulder Elbow Surg.

[35] Jones RB, Wright TW, Zuckerman JD (2016). Reverse total shoulder arthroplasty with structural bone grafting of large glenoid defects. J Shoulder Elbow Surg.

[36] Katz D, Valenti P, Kany J (2016). Does lateralisation of the centre of rotation in reverse shoulder arthroplasty avoid scapular notching? Clinical and radiological review of one hundred and forty cases with forty five months of follow-up. Int Orthop.

[37] Kennon JC, Lu C, McGee-Lawrence ME, Crosby LA (2017). Scapula fracture incidence in reverse total shoulder arthroplasty using screws above or below metaglene central cage: clinical and biomechanical outcomes. J Shoulder Elbow Surg.

[38] King JJ, Farmer KW, Struk AM, Wright TW (2015). Uncemented versus cemented humeral stem fixation in reverse shoulder arthroplasty. Int Orthop.

[39] Lädermann A, Denard PJ, Tirefort J (2017). Subscapularis- and deltoid-sparing vs traditional deltopectoral approach in reverse shoulder arthroplasty: a prospective case-control study. J Orthop Surg.

[40] Matsuki K, King JJ, Wright TW, Schoch BS (2018). Outcomes of reverse shoulder arthroplasty in small- and large-stature patients. J Shoulder Elbow Surg.

[41] Merolla G, Walch G, Ascione F (2018). Grammont humeral design versus onlay curved-stem reverse shoulder arthroplasty: comparison of clinical and radiographic outcomes with minimum 2-year follow-up. J Shoulder Elbow Surg.

[42] Mollon B, Mahure SA, Roche CP, Zuckerman JD (2016). Impact of glenosphere size on clinical outcomes after reverse total shoulder arthroplasty: an analysis of 297 shoulders. J Shoulder Elbow Surg.

[43] Mollon B, Mahure SA, Roche CP, Zuckerman JD (2017). Impact of scapular notching on clinical outcomes after reverse total shoulder arthroplasty: an analysis of 476 shoulders. J Shoulder Elbow Surg.

[44] Raiss P, Schnetzke M, Wittmann T (2019). Postoperative radiographic findings of an uncemented convertible short stem for anatomic and reverse shoulder arthroplasty. J Shoulder Elbow Surg.

[45] Romano AM, Oliva F, Nastrucci G (2019). Reverse shoulder arthroplasty patient personalized rehabilitation protocol. Preliminary results according to prognostic groups. Muscle Ligaments Tendons J.

[46] Schnetzke M, Preis A, Coda S (2017). Anatomical and reverse shoulder replacement with a convertible, uncemented short-stem shoulder prosthesis: first clinical and radiological results. Arch Orthop Trauma Surg.

[47] Simovitch R, Flurin P-H, Wright TW (2019). Impact of scapular notching on reverse total shoulder arthroplasty midterm outcomes: 5-year minimum follow-up. J Shoulder Elbow Surg.

[48] Valenti P, Sauzières P, Katz D (2011). Do less medialized reverse shoulder prostheses increase motion and reduce notching?. Clin Orthop Relat Res.

[49] Werner BC, Wong AC, Mahony GT (2018). Clinical outcomes after reverse shoulder arthroplasty with and without subscapularis repair: the importance of considering glenosphere lateralization. J Am Acad Orthop Surg.

[50] Werner BS, Ascione F, Bugelli G, Walch G (2017). Does arm lengthening affect the functional outcome in onlay reverse shoulder arthroplasty?. J Shoulder Elbow Surg.

[51] Zilber S, Camana E, Lapner P (2018). Reverse total shoulder arthroplasty using helical blade to optimize glenoid fixation and bone preservation: preliminary results in thirty five patients with minimum two year follow-up. Int Orthop.

[52] Lévigne C, Garret J, Boileau P (2011). Scapular notching in reverse shoulder arthroplasty: is it important to avoid it and how?. Clin Orthop Relat Res.

[53] Levy JC, Virani N, Pupello D, Frankle M (2007). Use of the reverse shoulder prosthesis for the treatment of failed hemiarthroplasty in patients with glenohumeral arthritis and rotator cuff deficiency. J Bone Joint Surg Br.

[54] Mulieri P, Dunning P, Klein S (2010). Reverse shoulder arthroplasty for the treatment of irreparable rotator cuff tear without glenohumeral arthritis. J Bone Jt Surg-Am.

[55] Teusink MJ, Pappou IP, Schwartz DG (2015). Results of closed management of acute dislocation after reverse shoulder arthroplasty. J Shoulder Elbow Surg.

[56] Corona K, Cerciello S, Ciolli G (2021). Clinical outcomes and joint stability after lateralized reverse total shoulder arthroplasty with and without subscapularis repair: a meta-analysis. J Clin Med.

[57] Romano AM, Braile A, Casillo P (2020). Onlay uncemented lateralized reverse shoulder arthroplasty for fracture sequelae type 1 with valgus/varus malunion: deltoid lengthening and outcomes. J Clin Med.

[58] Boileau P, Moineau G, Roussanne Y, Ohea K (2017). Bony increased offset-reversed shoulder arthroplasty (BIO-RSA). JBJS Essent Surg Tech.

[59] Erickson BJ, Frank RM, Harris JD (2015). The influence of humeral head inclination in reverse total shoulder arthroplasty: a systematic review. J Shoulder Elbow Surg.

[60] Lädermann A, Chiu JC-H, Cunningham G (2020). Do short stems influence the cervico-diaphyseal angle and the medullary filling after reverse shoulder arthroplasties?. Orthop Traumatol Surg Res.

[61] Chalmers PN, Boileau P, Romeo AA, Tashjian RZ (2019). Revision reverse shoulder arthroplasty. J Am Acad Orthop Surg.

[62] Ascione F, Domos P, Guarrella V (2018). Long-term humeral complications after Grammont-style reverse shoulder arthroplasty. J Shoulder Elbow Surg.

[63] Romano AM, Ascione T, Casillo P (2020). An evolution of shoulder periprosthetic infections management: MicroDTTect, bioactive glass and tantalum cones employment. J Clin Med.

[64] Guarrella V, Chelli M, Domos P (2019). Risk factors for instability after reverse shoulder arthroplasty. Shoulder Elb.

